# DNA Topoisomerases Are Required for Preinitiation Complex Assembly during *GAL* Gene Activation

**DOI:** 10.1371/journal.pone.0132739

**Published:** 2015-07-14

**Authors:** Morten Roedgaard, Jacob Fredsoe, Jakob Madsen Pedersen, Lotte Bjergbaek, Anni Hangaard Andersen

**Affiliations:** Laboratory of Genome Research, Department of Molecular Biology and Genetics, Aarhus University, C. F. Møllers Allé 3, DK-8000, Aarhus C, Denmark; Institute of Genetics and Molecular and Cellular Biology, FRANCE

## Abstract

To investigate the importance of topoisomerases for transcription of the galactose induced genes, we have studied the expression of *GAL1*, *GAL2*, *GAL7* and *GAL10* in *Saccharomyces cerevisiae* cells deficient for topoisomerases I and II. We find that topoisomerases are required for transcriptional activation of the *GAL* genes, but are dispensable for ongoing transcription, eliminating a role of the enzymes in transcriptional elongation. Furthermore, we demonstrate that promoter chromatin remodeling of the *GAL* genes is unaffected in the topoisomerase deficient strain. However, the cells fail to successfully recruit RNA polymerase II due to an inability of the TATA-binding protein (TBP) to bind to the TATA box in these promoters. We therefore argue that topoisomerases are required for accurate assembly of the preinitiation complex at the promoters of the *GAL* genes.

## Introduction

Several studies have demonstrated that transcription and DNA supercoiling are tightly coupled [1, 2]. The impact of transcription on DNA supercoiling has been explained by the “Twin-supercoiled-domain-model” [3], which implies that transcription by an RNA polymerase generates domains of positive and negative supercoiling in front of and behind the polymerase, respectively. These changes in superhelicity may eventually stop the advancing polymerase and/or perturb protein-DNA interactions if not removed or dispersed to other regions.

DNA topoisomerases solve topological problems arising during DNA metabolism. In *Saccharomyces cerevisiae* DNA superhelicity is primarily influenced by topoisomerase I (Top1) and topoisomerase II (Top2), encoded by the *TOP1* and *TOP2* gene, respectively [4]. Top1 removes helical tension by introducing a nick in one of the DNA strands, thus relieving superhelical tension by rotation of the cleaved strand around the intact strand. Top2 creates a transient double-stranded break in the DNA in order to transport another DNA duplex through the break [4]. Thus, both enzymes are able to relax supercoiled DNA, but they show differences in their substrate preferences, where Top1 is faster than Top2 in relaxation of naked DNA, whereas the opposite is the case when nucleosomal DNA is relaxed [5].

Chromatin structure adds another layer of complexity to DNA supercoiling. Approximately 80% of the genome is covered by nucleosomes in yeast [6], and nucleosomes influence transcription as they release and absorb negative superhelicity by dissociation and re-association with DNA, respectively [7]. In support of this, topoisomerases have been demonstrated to affect nucleosome dynamics. Thus, an early study showed a requirement of either Top1 or Top2 for proper chromatin assembly [8], and more recently a genome wide study demonstrated a direct requirement of Top1 for efficient nucleosome disassembly at gene promoters [9].

It has recently been suggested that chromatin is able to adapt to changes in DNA superhelicity by a slight conformational change, which is reverted upon relaxation by either Top1 or Top2 [5]. This implies that the chromatin fiber is a torsionally resilient structure, which can act as a topological buffer and facilitate dissipation of topological strain [10–12]. In addition to this, gathering evidence points to the conclusion that supercoiling is a dynamic entity, which is able to spread from the site of generation to far-reaching regions, thereby having long ranging effects [1, 12]. In eukaryotes, a change in DNA superhelicity may thus exert an additional effect on transcription via changes at the chromatin level.

Several studies have established a role of topoisomerases in transcription and transcriptional regulation. Accordingly, a genome-wide study in yeast showed a preferential localization of the enzymes to intergenic regions, i.e. promoter regions, of highly transcribed genes [13, 14], and Top1 and Top2 were found to act redundantly to enhance the recruitment of RNA polymerase II [13]. Other yeast studies have shown up- or downregulation of specific genes in the absence of either Top1 or Top2 activity, demonstrating roles of the individual enzymes in transcriptional regulation [15, 16]. Furthermore, transcription of highly expressed genes were shown to require both topoisomerase I and II in human cells, whereas genes with lower transcription managed with only topoisomerase I, demonstrating the importance of topoisomerases in gene regulation [17].

A recent study from our laboratory combined microarray gene expression analyses and single gene studies to investigate the role of topoisomerases for global gene expression [15]. Topoisomerases were found to have a major impact on transcription of a subset of genes, characterized by highly regulated transcription initiation. For the inducible *PHO5* gene we demonstrated that topoisomerases were required during transcriptional activation, but not for reinitiation and transcription elongation. In the absence of topoisomerase activity, the Pho4 transcription factor failed to bind to the promoter, thus inhibiting eviction of nucleosomes from the promoter region.

In the present work we have studied transcription of the galactose inducible genes, *GAL1*, *GAL2*, *GAL7*, and *GAL10* to investigate if topoisomerases have a similar effect on the transcriptional activation of these genes. As for the *PHO5* gene, we find that topoisomerases are essential for activation of the *GAL* genes but not for ongoing transcription. However, we discover that nucleosome removal from the promoters is unperturbed during transcriptional activation of the *GAL* genes in a strain lacking functional topoisomerases, but the strain displays faulty RNA polymerase II recruitment to the *GAL* gene promoters. In correlation with this, we find that the TATA-binding protein (TBP) fails to bind to the TATA box in these promoters, suggesting an involvement of topoisomerases in an early step of preinitiation complex assembly. Thus, although the overall effect of topoisomerase deficiency is the same for different inducible genes, the specific step, where the enzymes exert their effect, may vary from gene to gene.

## Materials and Methods

### Yeast strains and growth conditions

All *S*. *cerevisiae* strains used are derivatives of W303a and are listed in Table 1. For galactose induction experiments, yeast strains were grown to a density of ~10^7^ cells/ml at 25°C in YP with 2% raffinose and arrested in G1 with α-factor (Lipal Biochem, Zürich, Switzerland). Top2 was subsequently inhibited for 15 min at 37°C before galactose was added to a final concentration of 2% for induction of the galactose responsive genes. A sample was taken just prior to addition of galactose to serve as an uninduced control.

**Table 1:** *S*. *cerevisiae* strains used in this study.

Strain	Genotype	Source
Ay-120	MATa *ade2*-1 *trp1*-1 *his3*-11 *his3*-15 *ura3*-1 *leu2*-3 *leu2*-112 *canI*-100 Cir0	R. Rothstein (W303)
Ay-109	Ay-120 with *top1*::*NAT top2*-*1* ^*ts*^	[15]
Ay-127	Ay-120 with *top2*-*1* ^*ts*^	[15]
Ay-161	Ay-120 with *top1*::*NAT*	[15]
Ay-375	Ay-120 with *TOP1*-13xcMyc-*TRP*	This study
Ay-386	Ay-120 with *TOP2*-13xcMyc-*TRP*	This study
Ay-424	Ay-120 with *gal80*::*URA3*	This study
Ay-425	Ay-120 with *top1*::*NAT top2*-1^ts^, *gal80*::*URA3*	This study
Ay-483	Ay-120 with 3xHA-TBP-*URA3*	K. Struhl (YLK4)
Ay-484	Ay-120 with *top1*::*NAT top2-1* ^*ts*^, 3xHA-TBP-*URA3*	This study

### mRNA extraction and qPCR

**Fig 1:**
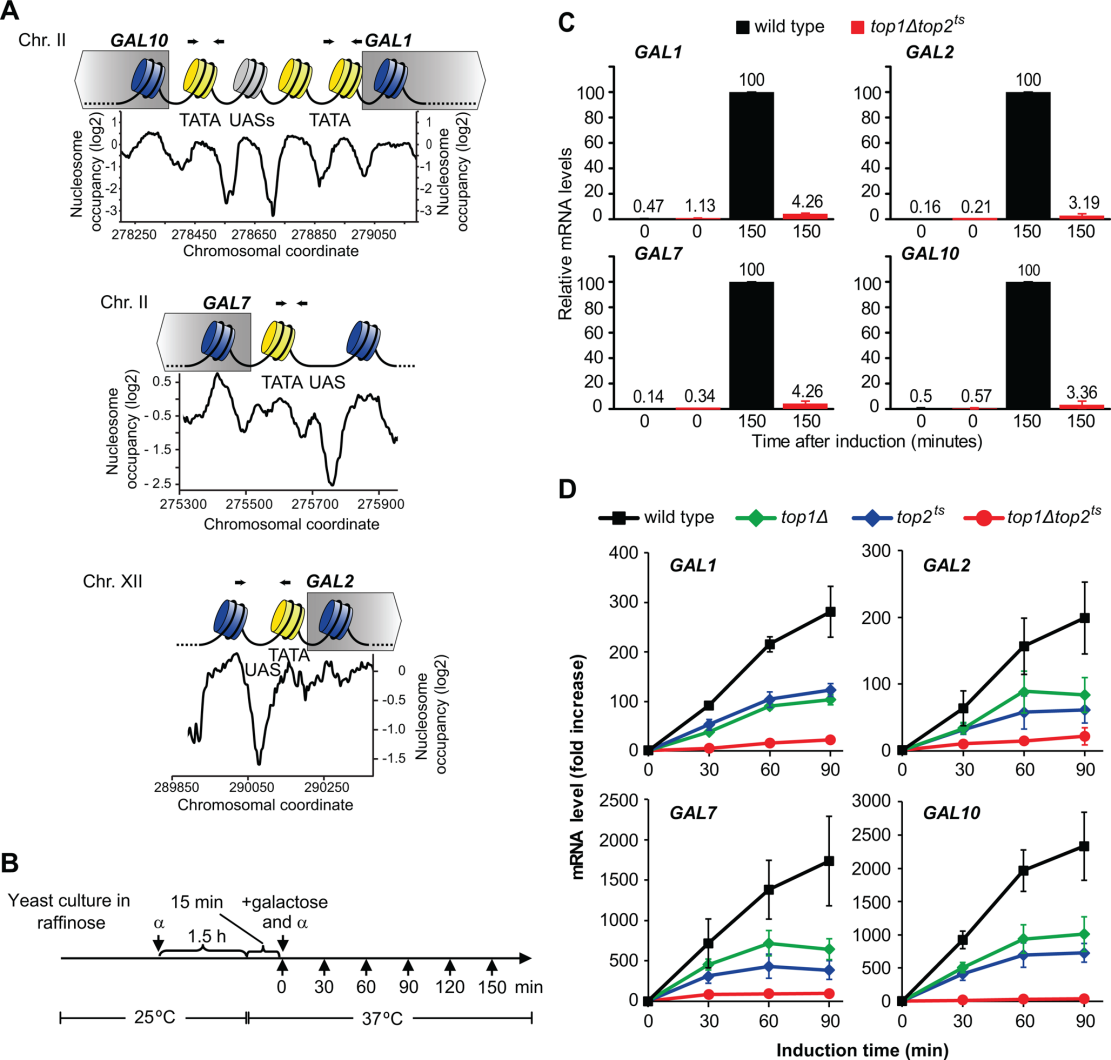
*GAL* gene transcription requires topoisomerase activity. (A) Organization of the *GAL* genes. *GAL1* and *GAL10* are located on Chromosome II (Chr. II) and share a promoter with two UASs and three nucleosomes. The *GAL7* promoter, located immediately after the *GAL10* open reading frame on Chr. II, contains a single UAS and a single nucleosome. The *GAL2* promoter is similar to the *GAL7* promoter, but is located on Chr. XII. Promoter nucleosomes are illustrated in yellow. A promoter nucleosome occupancy profile [6] is shown below each gene (black line) indicating chromosomal coordinates of nucleosomes. Primers used in ChIP experiments are indicated by black arrows. UAS, Upstream Activating Sequence. TATA, TATA box. (B) Experimental setup. Cells were grown at 25°C in raffinose media (de-repressive conditions), and α-factor was added to arrest cells in G1. After 1.5 hours, cells were shifted to the restrictive temperature. After Top2 inactivation for 15 minutes, cells were treated with galactose to induce the *GAL* genes, α-factor was added again to keep cells in G1, and samples were collected at the indicated time points. α, α-factor. (C) Induction of *GAL1*, *GAL2*, *GAL7*, and *GAL10* in wild type and *top1Δtop2* ^*ts*^ cells. Cells were treated as illustrated in (B), and samples were collected 0 and 150 minutes after galactose treatment. mRNA was isolated, and the levels of the individual *GAL* genes and two control genes (*GAPDH* and *ACT1*) were quantified by qPCR. mRNA levels of the *GAL* genes were calculated relative to the mean of the mRNA levels for *GAPDH* and *ACT1* for each time point and the value obtained in wild type at the latest time point was set to 100. Numbers indicate relative mRNA levels. Averages from two individual experiments are shown with error bars representing ± one standard deviation. (D) Induction of *GAL1*, *GAL2*, *GAL7*, and *GAL10* in wild type, *top1Δ*, *top2* ^*ts*^, and *top1Δtop2* ^*ts*^ cells. Cells were treated as shown in (B), and samples were collected at the indicated time points for qPCR measurements of *GAL* gene mRNA levels. mRNA levels are presented as fold increase relative to the level at time point 0. Averages from three individual experiments are shown with error bars representing ± one standard deviation.

**Fig 2:**
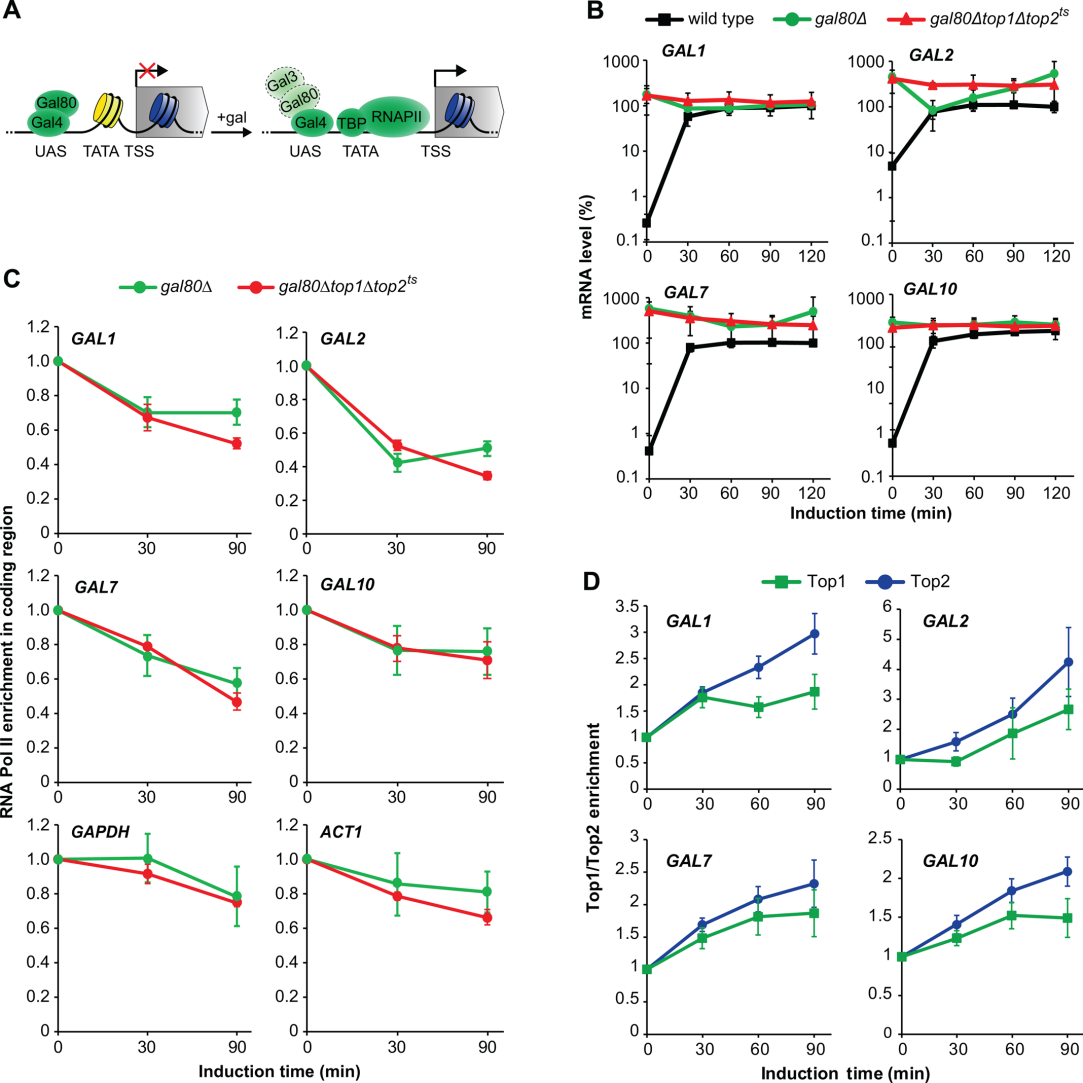
Topoisomerase activity has a direct role in *GAL* gene activation but is not required for transcriptional elongation and reinitiation. (A) Overview of promoter changes during transcriptional induction of the *GAL* genes. (*left*) In raffinose, the *GAL* gene promoter is covered by nucleosomes except at the UAS, which binds Gal4 having its activation domain blocked by Gal80. (*right*) Upon galactose addition, Gal3 binds Gal80, leaving the activation domain of Gal4 free to bind chromatin remodelers. Subsequent removal of promoter nucleosomes allows recruitment of TBP and RNA polymerase II. UAS, Upstream Activating Sequence. TATA, TATA box. TSS, Transcription Start Site. gal, galactose. Light coloring and dashed borders of Gal3 and Gal80 indicate that the enzymes do not block the Gal4 activation domain, either due to dissociation or rearrangement of the complex. (B) Time course experiment of *GAL1*, *GAL2*, *GAL7*, and *GAL10* transcription in wild type, *gal80Δ*, and *gal80Δtop1Δtop2* ^*ts*^ cells. Cells were treated as in Fig 1B, and mRNA levels of the individual genes were quantified by qPCR, normalized to the wild type level at the latest time point (set to 100%), and presented on a log10-scale. The average from two individual experiments is shown, and error bars represent ± one standard deviation. (C) Time course experiment with ChIP analysis of RNA polymerase II enrichment in the coding regions of the *GAL* genes and two control genes, *GAPDH* and *ACTI*, following transcriptional activation. Cells were treated as in Fig 1B, and ChIP was performed with antibodies recognizing the C-terminal domain of the Rpb1 subunit of RNA polymerase II. RNA polymerase II binding levels were normalized relative to the binding at the 0 min time point (set to 1). (D) Time course experiment with ChIP analysis of Top1 and Top2 enrichment in the promoters of the *GAL* genes following transcriptional activation. Cells expressing the endogenous Top1 or Top2 enzymes fused to a cMyc tag were treated as described in Fig 1B, and ChIP was performed with antibodies recognizing the cMyc tag. Top1 and Top2 binding levels were normalized as in (C). In (C) and (D) averages from three individual experiments are shown, and error bars represent ± one standard deviation. Positions of primers used in the ChIP experiments for the individual *GAL* genes are indicated with arrows in Fig 1A and presented in Table 2.

For analysis of transcription levels by qPCR, cells were grown as described above, and samples of 2x10^7^ cells were collected at the indicated time points. RNA was purified by use of RNeasy (Invitrogen, Carlsbad, CA), and cDNA was made by SuperScript II RT-PCR (Invitrogen, Carlsbad, CA) using oligo dT primers (Figs 1C and 2B) or by QuantiTect Reverse Transcription Kit (Qiagen) using random primers (Fig 1D). mRNA levels were quantified by Real-time PCR performed with HOT FIREPol EvaGreen qPCR Mix Plus (Solis Biodyne, Tartu, Estonia) using a Stratagene MX3000 (Agilent, Santa Clara, CA) and normalized to the ACT1 and GAPDH mRNA levels. Primer sequences are listed in Table 2.

**Table 2:** Primers used in this study.

Name	Sequence	Comment
**AP 487**	CACCAACTGTTTGGCTCCAT	RT-qPCR of *TDH1* (*GAPDH*)
**AP 488**	TAGCAGCACCGGTAGAGGAT	RT-qPCR of *TDH1* (*GAPDH*)
**AP 1820**	CCATAATGCCTCCTATATTTAGCCTTT	ChIP at TEL06R (normalization)
**AP 1821**	TCCGAACGCTATTCCAGAAAGT	ChIP at TEL06R (normalization)
**AP 2109**	GCCTTCTACGTTTCCATCCA	RT-qPCR of *ACT1*
**AP 2110**	GGCCAAATCGATTCTCAAAA	RT-qPCR of *ACT1*
**AP 2161**	AACAAACATTTCGCAGGCTA	ChIP in the promoter of *GAL2*
**AP 2162**	TATTCTTGATGATAATTGAA	ChIP in the promoter of *GAL2*
**AP 2165**	TTCCGACCTGCTTTTATATC	ChIP in the promoter of *GAL7*
**AP 2166**	ACAGTGTTCACAAAATAGCC	ChIP in the promoter of *GAL7*
**AP 2190**	AGCTGCATAACCACTTTAAC	ChIP in the promoter of *GAL1*
**AP 2191**	GACGTTAAAGTATAGAGGTA	ChIP in the promoter of *GAL1*
**AP 2192**	GGCATTACCACCATATACAT	ChIP in the promoter of *GAL10*
**AP 2193**	GAAAGTTCCAAAGAGAAGGT	ChIP in the promoter of *GAL10*
**AP 2359**	CGTTGCTTTAGCTGTTGTT	RT-qPCR of *GAL1*
**AP 2360**	CTGATCCATACCGCCATT	RT-qPCR of *GAL1*
**AP 2361**	TTGGCCTGGATGATTCCT	RT-qPCR of *GAL2*
**AP 2362**	AGCGCCCAAAAGTAAACA	RT-qPCR of *GAL2*
**AP 2363**	CCCAGTATGGAACAACAAC	RT-qPCR of *GAL7*
**AP 2364**	CTGATTTGTTTGCCGATTAC	RT-qPCR of *GAL7*
**AP 2365**	ACCAGAAGCTTTGCAGAA	RT-qPCR of *GAL10*
**AP 2366**	AAGGTTTGTGTCGTGAGT	RT-qPCR of *GAL10*

### ChIP and Western Blot

ChIP was performed with 2.5x10^8^ cells as described previously [15] with minor modifications. Thus, wash of antibody-coupled beads after incubation with extract was performed two times with Lysis buffer (50 mM Hepes, pH 7.5, 140 mM NaCl, 1 mM EDTA, pH 8, 1% Triton X-100, 0.1% Natriumdeoxycholate and protease inhibitors), one time with Wash buffer (10 mM Tris-HCl, pH 8, 500 mM NaCl, 1 mM EDTA, pH 8, 0.5% NP-40, 0.5% Natriumdeoxycholate and protease inhibitors), and one time with TE buffer (Tris-HCl, pH 7.5, mM EDTA). Histone H3 was precipitated with monoclonal antibodies recognizing the H3 C-terminal tail (ab1791 available from Abcam, Cambridge, UK), Gal4 was precipitated with a polyclonal anti-Gal4 antibody (ab1396 available from Abcam, Cambridge, UK) and RNA polymerase II was precipitated using a monoclonal antibody against the C-terminal domain of the RPB1 subunit (ab5408 available from Abcam, Cambridge, UK). For ChIP of RNA polymerase II, an extra washing step was included with Lysis500 (Lysis buffer containing 500 mM NaCl) after before wash with the Wash buffer. For ChIP of 3xHA-tagged TBP, monoclonal antibodies against the HA epitope tag were used (Santa Cruz), whereas ChIP of cMyc tagged Top1 or Top2 was performed with monoclonal antibodies targeting the cMyc epitope tag (Santa Cruz). Enrichment was calculated as 2^(CT IP – CT beads)/CT Input^, and was normalized to the enrichment in a telomeric region (TEL06R). The 0 min time point was set to 1. Sequences of the primers used in the ChIP experiments are listed in Table 2. For each gene, the same primer set was used for ChIP of H3, the RPB1 subunit of RNA polymerase II, TBP, Top1, Top2, and Gal4. The resolution of the individual ChIP assays was approximately 500 bp. For Western blotting of TBP, proteins were precipitated from wild type and top1Δtop2^ts^ cells expressing HA tagged TBP by trichloroacetic acid. Proteins were subjected to sodium dodecyl sulphate polyacrylamide gel electrophoresis, and western blotting was performed with antibodies targeting the HA epitope tag on TBP (Santa Cruz) and anti Mcm2 antibodies (Santa Cruz).

## Results

### Topoisomerase activity is required for *GAL* gene transcription

The *GAL* genes are a group of genes, which is induced when galactose is used as the carbon source in *S*. *cerevisiae*. To investigate, how topoisomerase deficiency influences transcription of this group of genes, we have studied transcription of *GAL1*, *GAL2*, *GAL7* and *GAL10* in an *S*. *cerevisiae* strain having a deletion of the *TOP1* gene and a temperature-sensitive mutation in the *TOP2* gene (*top10Δtop2* ^*ts*^). A schematic presentation of the individual *GAL* genes is shown in Fig 1A. In an earlier study we found that absence of topoisomerases dramatically inhibited the transcription of inducible genes, including the *GAL* genes [15]. To establish if lack of topoisomerases causes a true inhibition of *GAL* gene transcription or merely a kinetic delay, we performed an experiment with wild type and *top1Δtop2* ^*ts*^ cells as outlined in Fig 1B, where transcription was followed for an extensive period of time. To avoid genome-wide effects of topological challenges caused by replication [14, 18] as well as abortive mitosis due to lack of Top2 activity, the cells were kept in G1 throughout the experiment by treatment with α-factor. After inactivation of Top2 by transfer of cells to the restrictive temperature of 37°C, the *GAL* genes were activated by addition of galactose. Cells were collected before transfer to inducible conditions (“0”) and after an induction period of 150 min, and mRNA levels were determined by qPCR. In *top1Δtop2* ^*ts*^ cells the induction levels of all four *GAL* genes remained very low even after 150 minutes under inducing conditions relative to the levels obtained in wild type cells (Fig 1C). Thus, the absence of topoisomerases does not result in a kinetic delay in *GAL* gene transcription. Rather, the enzymes are required for transcription of the genes *per se*.

The observed lack of *GAL* gene transcription in *top1Δtop2* ^*ts*^ cells suggests that topoisomerase relaxation activity is required for transcription. Alternatively, one of the enzymes could play a more specific, although essential role for *GAL* gene transcription, in which case no transcription would be observed in cells lacking this enzyme. To differentiate between these two possibilities, we compared *GAL* gene transcription in *top1Δ*, *top2* ^*ts*^ and *top1Δtop2* ^*ts*^ cells using the experimental setup outlined in Fig 1B. Although *top1Δ* and *top2* ^*ts*^ cells showed reduced *GAL* gene transcription relative to wild type cells, the level in each single mutant was significantly increased compared to the level in the double mutant (Fig 1D). This demonstrates that *GAL* gene transcription is sensitive to topoisomerase dosage. The result therefore strongly suggests that *GAL* gene transcription requires DNA relaxation activity, which is the only common activity of Top1 and Top2.

### Topoisomerases are directly required for *GAL* gene activation but are dispensable for transcriptional elongation and reinitiation

When yeast cells are grown in media containing raffinose, the *GAL* genes are in a “primed” or de-repressed state, ready to undergo rapid induction upon galactose addition [19]. In this state, the transcription factor Gal4 is bound to the Upstream Activating Sequence (UAS) in the promoter region. However, the protein domain responsible for recruitment of factors involved in transcriptional activation is blocked by Gal80 through protein-protein interactions (Fig 2A, *left*) [20, 21]. When galactose is added to the media, the activation domain on Gal4 becomes accessible for binding of chromatin remodeling factors. This has been suggested to take place either by a direct dissociation of Gal80 due to interaction with Gal3 [22, 23] or by formation of a complex between Gal4, Gal80 and Gal3, which leaves the domain accessible for other interactions [24, 25]. Binding of chromatin remodeling factors ensures nucleosome eviction from the promoter region and exposure of the TATA box, thus paving the way for transcriptional activation (Fig 2A, *right*) [24, 26]. To investigate, whether topoisomerases were required for transcriptional activation of the *GAL* genes or for continued transcription, we took advantage of the fact that deletion of *GAL80* leads to constitutive expression of the *GAL* genes due to elimination of the need for external stimuli. Wild type, *gal80Δ*, as well as *gal80Δtop1Δtop2* ^*ts*^ cells were therefore cultured as shown in Fig 1B, and samples were withdrawn at the indicated time points and analyzed for *GAL* gene mRNA levels. In contrast to wild type cells *gal80Δ* and *gal80Δtop1Δtop2* ^*ts*^ cells displayed high mRNA levels of the *GAL* genes under de-repressive conditions at the “0” minutes time points (reflecting the situation at the permissive temperature for *top2* ^*ts*^) (Fig 2B). Interestingly, following transfer to inducible conditions at the restrictive temperature, *gal80Δtop1Δtop2* ^*ts*^ cells still accumulated mRNA at a level comparable to *gal80Δ* cells and similar to fully induced wild type cells. This demonstrates that once activation has taken place, continued transcription including transcription elongation is independent of topoisomerase activity.

To verify that transcription elongation took place to the same extent in *gal80Δ* and *gal80Δtop1Δtop2* ^*ts*^ cells we investigated the level of RNA polymerase II in the coding region of the *GAL* genes in the two strains. This was performed by chromatin immunoprecipitation (ChIP) with antibodies against the C-terminal domain of the RPBI subunit of RNA polymerase II (Fig 2C). As expected, the level of RNA polymerase II was similar in the coding regions of the *GAL* genes in the two strains, consistent with a topoisomerase independency of *GAL* gene transcription elongation. A drop in RNA polymerase II enrichment of about 50% was seen in both strains during the experiment. A drop was also seen in two control genes, *GAPDH* and *ACTI*, and probably reflects the transfer of cells to 37°C. Taken together, the experiments demonstrate that topoisomerases are required during the initial activation of the *GAL* genes, whereas transcription elongation and re-initiation can occur in the absence of topoisomerase activity, as was observed earlier for the *PHO5* gene [15].

The observed requirement of topoisomerase activity during *GAL* gene activation can be caused by a direct need for DNA relaxation activity in the *GAL* gene promoters. However, relaxation activity could also be required indirectly in a separate step essential for *GAL* gene activation. If the enzymes exert a direct function to relax promoter regions during *GAL* gene activation, we expect a recruitment of Top1 and Top2 to the promoter regions during activation. To investigate if this took place, yeast strains expressing either Top1 or Top2 fused to a cMyc epitope tag were generated, and chromatin immunoprecipitation (ChIP) was performed with antibodies targeting the cMyc epitope tag. Top1 and Top2 were enriched approximately 2 and 3 fold, respectively, in the individual *GAL* gene promoters (Fig 2D), demonstrating a recruitment of both topoisomerases to the promoter regions upon induction. Taken together, the results suggest that Top1 and Top2 directly bind to the promoter regions to ensure that the topological state is optimal for efficient *GAL* gene activation.

### Eviction of nucleosomes from the *GAL* gene promoters during activation occurs independent of topoisomerases

**Fig 3:**
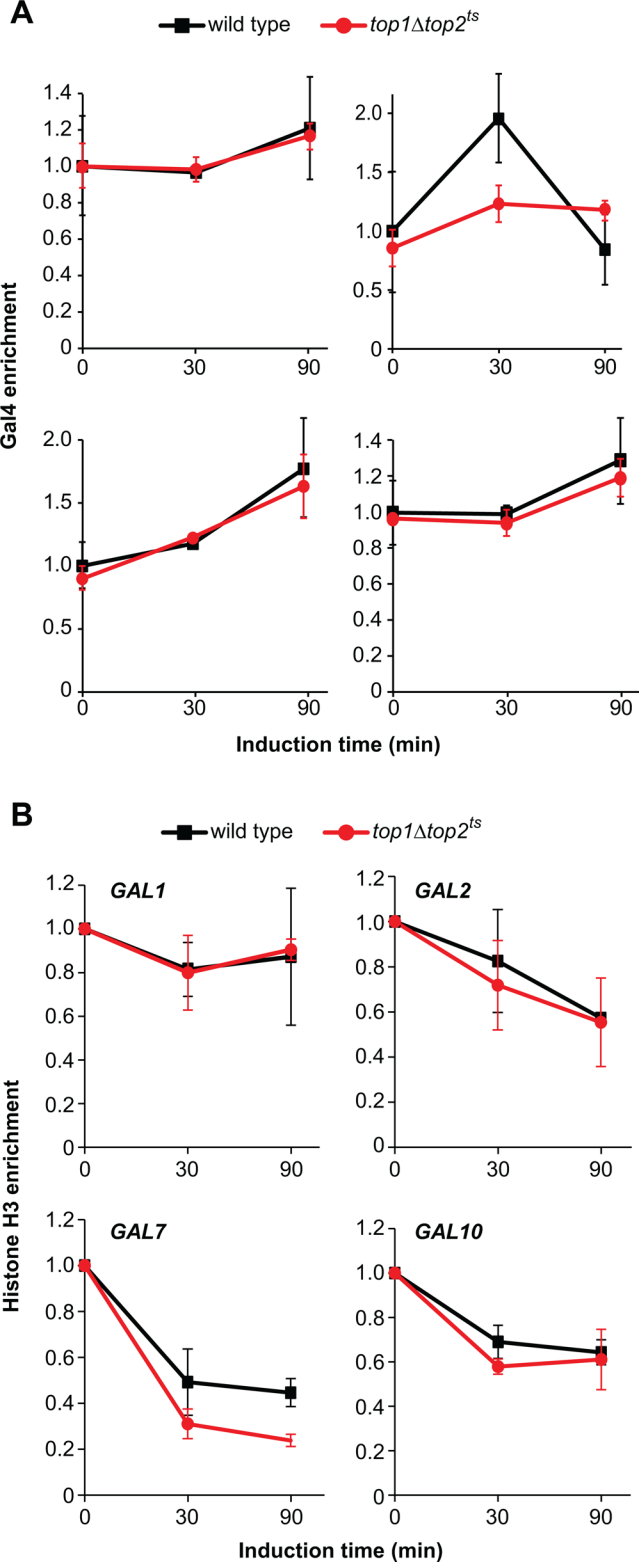
Promoter chromatin remodeling is not affected by lack of topoisomerases. (A) ChIP analysis of Gal4 binding in the *GAL* gene promoters of wild type and *top1Δtop2* ^*ts*^ cells following transcriptional activation. Experimental setup was as described for Fig 1B, and ChIP was performed using antibodies against Gal4. Gal4 binding levels in the *GAL* gene promoters were normalized to the binding under de-repressed conditions in wild type at the 0 min time point (set to 1). (B) ChIP analysis of nucleosome removal from *GAL* gene promoters of wild type and *top1Δtop2* ^*ts*^ cells following transcriptional activation. ChIP was performed using antibodies targeting histone H3. H3 binding levels in the *GAL* gene promoters were normalized relative to the binding under uninduced conditions at the 0 min time point (set to 1). Averages from three (A) or two (B) individual experiments are shown, and error bars represent ± one standard deviation. Positions of primers used in the ChIP experiments for the individual *GAL* genes are indicated with arrows in Fig 1A and presented in Table 2.

We next addressed, during which step in the activation process topoisomerases were required. A prerequisite for induction of the galactose responsive genes is removal of nucleosomes from the promoter regions to allow access of essential transcription factors (Figs 1A and 2A) [27]. Since the presence of Gal4 in the promoter region is essential for this step, we first used ChIP with antibodies against Gal4 to investigate whether or not this factor remained bound in *top1Δtop2* ^*ts*^ cells. For all *GAL* genes, the level of Gal4 in the promoter region was similar in wild type and *top1Δtop2* ^*ts*^ cells (Fig 3A). Thus, Gal4 remains bound in the absence of topoisomerases leaving the fundament for chromatin remodeling intact in *top1Δtop2* ^*ts*^ cells.

To investigate, whether nucleosome removal *per se* was perturbed in *top1Δtop2* ^*ts*^ cells, we next used ChIP with antibodies targeting histone H3 to measure the nucleosomal occupancy in the promoter regions of the *GAL* genes during galactose induction. Nucleosomes were removed with equal kinetics and to a similar extent in both wild type and *top1Δtop2* ^*ts*^ cells (Fig 3B). Thus, in contrast to our earlier observation with the inducible *PHO5* gene [15], nucleosome removal from the *GAL* gene promoters occurs independent of topoisomerase activity.

### *GAL* gene activation requires topoisomerases for preinitiation complex assembly

**Fig 4:**
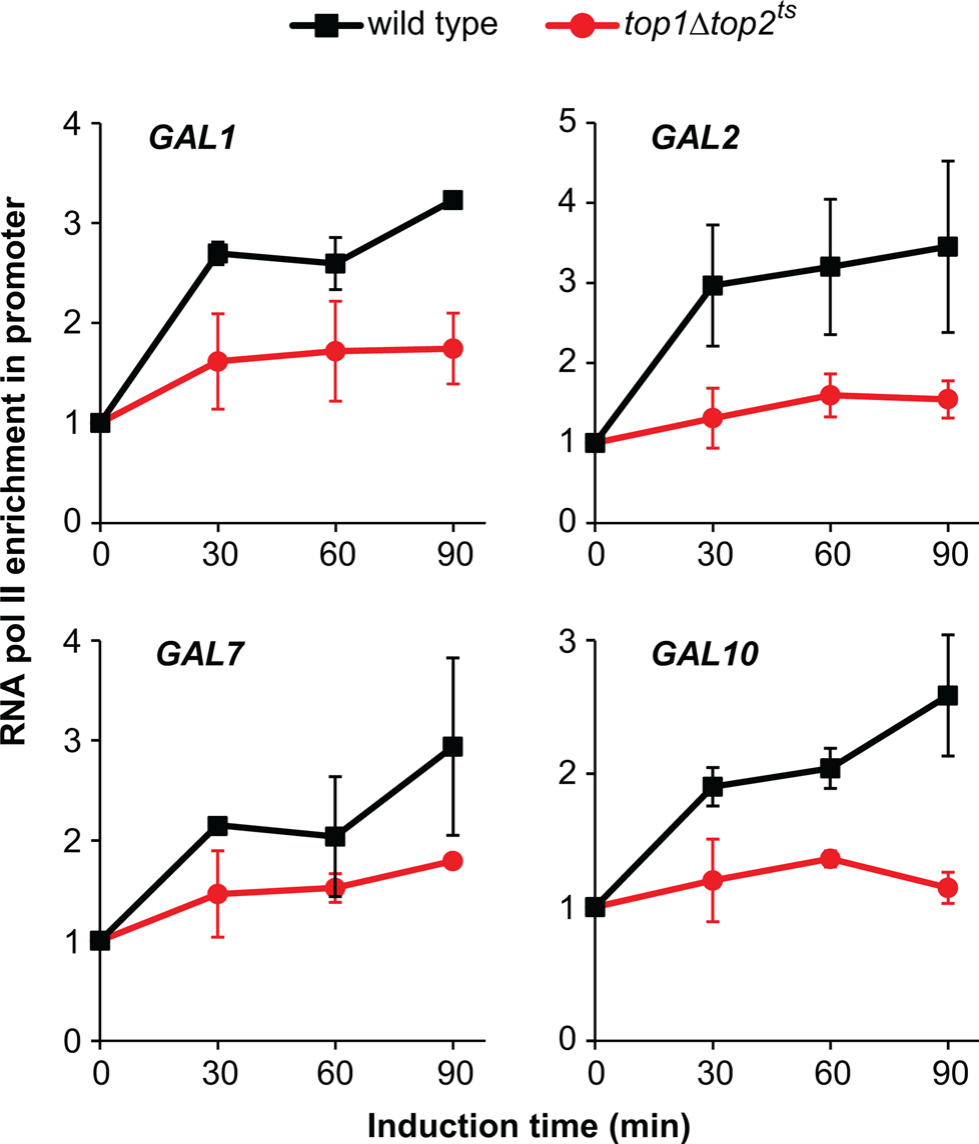
Topoisomerase activity is required for RNA polymerase II recruitment. ChIP analysis of RNA polymerase II enrichment in *GAL* gene promoters of wild type and *top1Δtop2* ^*ts*^ cells following transcriptional activation. Experimental setup was as described for Fig 1B, and ChIP was performed using antibodies targeting RNA polymerase II. RNA polymerase II binding levels were normalized relative to the binding under uninduced conditions at the 0 min time point (set to 1). Averages from two individual experiments are shown, and error bars represent ± one standard deviation. Positions of primers used in the ChIP experiments for the individual *GAL* genes are indicated with arrows in Fig 1A.

The step following eviction of nucleosomes from the promoters in transcriptional activation of the *GAL* genes is assembly of the preinitiation complex [19]. A principal part of this assembly is the recruitment of RNA polymerase II. In order to study the recruitment of RNA polymerase II to the promoters of the *GAL* genes, ChIP was performed using antibodies against the C-terminal domain of the *RPB1* subunit of RNA polymerase II. As expected, an increase in RNA polymerase II occupancy in the promoter regions of the *GAL* genes was observed in wild type cells (Fig 4). Conversely, very little or no RNA polymerase II enrichment was seen in the *GAL* gene promoters in *top1Δtop2* ^*ts*^ cells, demonstrating a requirement of topoisomerases for the recruitment of RNA polymerase II to the *GAL* gene promoters.

**Fig 5:**
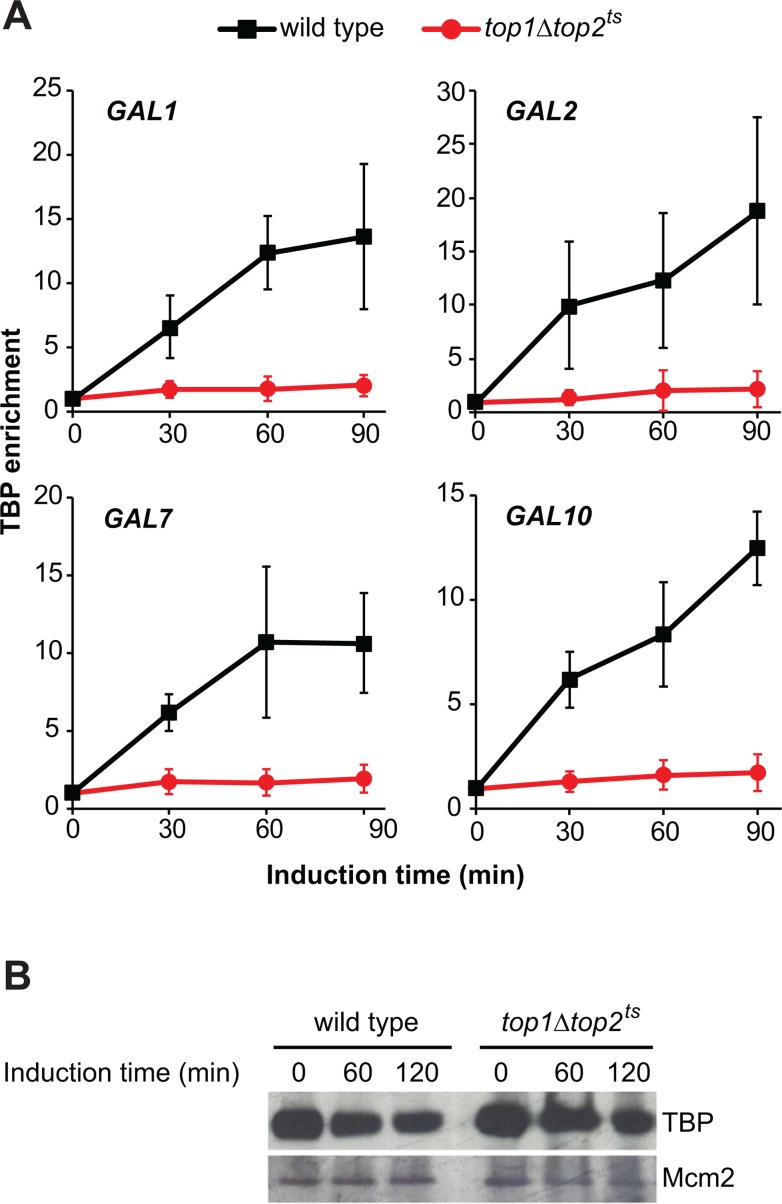
TBP binding to the TATA box requires topoisomerase activity. (A) ChIP analysis of TBP enrichment in *GAL* gene promoters of wild type and *top1Δtop2* ^*ts*^ cells following transcriptional activation. Experimental setup was as described for Fig 1B. ChIP was performed on cells having the endogenous TBP protein fused to a HA epitope tag using anti-HA antibodies. TBP binding levels were normalized relative to the binding under uninduced conditions at the 0 min time point (set to 1). Averages from three individual experiments are shown, and error bars represent ± one standard deviation. Positions of primers used in the ChIP experiments for the individual *GAL* genes are indicated with arrows in Fig 1A. (B) TBP protein levels in wild type and *top1Δtop2* ^*ts*^ cells. Cells used in (A) were treated as in Fig 1B, and samples taken at the indicated time points were processed for Western Blot analysis with antibodies targeting the HA tag on TBP. Mcm2 was used as loading control.

Binding of the TATA-binding protein (TBP) to the TATA box is the first step in the assembly of the preinitiation complex [28]. In order to investigate the role of topoisomerases in the binding of TBP to the TATA box in the promoter regions of the *GAL* genes, ChIP was again performed, this time with antibodies targeting an HA-tag N-terminally fused to TBP. As expected, an increase in TBP enrichment was observed in the promoters of the *GAL* genes in wild type cells (Fig 5A). However, no significant enrichment was seen in *top1Δtop2* ^*ts*^ cells. Taken together with the observation that the cellular level of TBP was similar in wild type and topoisomerase deficient cells, (Fig 5B), this demonstrates a requirement of topoisomerases for TBP binding. Based on our results, we conclude that *GAL* gene activation requires topoisomerases either directly for TBP binding to the TATA box, or in a step between nucleosome eviction and TBP binding.

## Discussion

Our findings have revealed that topoisomerase activity is required for transcriptional activation of the *GAL* genes, whereas ongoing transcription can occur in the absence of Top1 and Top2. Furthermore, we demonstrate that topoisomerases are required for TBP binding to the TATA box, or in a step downstream of nucleosome eviction, but upstream of TBP binding. We have previously shown that topoisomerases are required for transcriptional activation of the phosphate regulated *PHO5* gene, where the enzymes are necessary for binding of the transcription factor Pho4 to the promoter region [15]. In the present study, lack of topoisomerase activity does not influence the *GAL* gene induction pathway, as wild type and *top1Δtop2* ^*ts*^ cells display equal nucleosome promoter clearing (Fig 3). The disparities in topoisomerase requirement between the *PHO5* gene and the *GAL* genes are probably reflected in the dissimilarities in the induction pathways. In the case of *PHO5*, the Pho4 transcription factor has to bind the *PHO5* promoter in order for nucleosome remodeling to occur [29]. In contrast, the corresponding Gal4 transcription factor, which is required for chromatin remodeling in the *GAL* system, is already bound to the promoter, but its chromatin remodeler recruiting domain is blocked by Gal80 [21]. Thus, this step in chromatin remodeling does not rely on novel protein-DNA interactions as it does in the *PHO5* system.

Interestingly, no RNA polymerase II or TBP enrichment is observed in the promoters of the *GAL* genes in *top1Δtop2* ^*ts*^ cells during transcriptional activation (Figs 4 and 5), whereas topoisomerases become dispensable once the *GAL* genes are activated (Fig 2). This indicates that transcriptional activation is fully dependent on topoisomerase activity although reinitiation and transcription elongation can take place in the already activated genes independent of topoisomerases. One explanation for this discrepancy could be that the half-lives of the mRNAs in question are very high, making it impossible to accurately assess, whether transcription in the *gal80∆top1Δtop2* ^*ts*^ mutant is deregulated upon transfer to the restrictive temperature. However, as the half-lives of the mRNA’s from *GAL1*, *GAL2*, *GAL7*, and *GAL10* are 18, 49, 27, and 20 minutes, respectively [30], and we measure transcript levels 135 min after transfer of the cells to the restrictive temperature for *top2* ^*ts*^, we find this explanation unlikely. A more plausible explanation is that after the RNA polymerase has escaped from the preinitiation complex, some of the general transcription factors remain in the promoter region, creating a reinitiation scaffold [31], which can persist even under topological conditions, where one or more of the factors would be unable to bind individually. Furthermore, several *GAL* genes have been shown to exhibit gene looping [32, 33], where the promoter and termination regions are brought into close proximity. Gene looping allows for easy RNA polymerase shuttling from the termination region to the promoter, and is believed to keep the promoter chromatin free and primed for reinitiation, which may eliminate the requirement for topoisomerases.

Transcriptional activation of a gene is a complicated process involving a plethora of proteins. The DNA superhelical changes occurring in topoisomerase deficient strains will most likely affect the activation process in a step involving protein-DNA interactions. As the nucleosomes bound at the *GAL* gene promoters are removed with equal kinetics in both wild type and *top1Δtop2* ^*ts*^ cells (Fig 3), but TBP is unable to bind to the promoter in topoisomerase deficient cells, the requirement for topoisomerase activity has to be found between these two steps. Following nucleosome depletion, different co-activators, including Mediator and SAGA, bind at *GAL* gene promoters through interaction with Gal4 [34, 35]. These co-activators then assist in the recruitment of TBP to the TATA box through direct interactions [20]. The step requiring topoisomerase activity can therefore either be binding of TBP or one of its co-activators. However, we find it unlikely that binding of Mediator and SAGA is influenced by lack of topoisomerase activity, since these co-activators have been suggested to act as bridging proteins between Gal4 and TBP [20]. Rather, we expect that the DNA interaction of TBP will be impeded in topoisomerase deficient strains, where the global superhelical level is shifted towards a more positive state [12, 15, 36]. To this end, earlier studies have demonstrated that TBP binding is supercoiling sensitive [37–39]. This is supported by the earlier finding that TBP binding extensively distorts the DNA helix by introducing a 90° kink in the DNA, causing a slight underwinding of the DNA [39, 40]. Furthermore, an *in vitro* study has demonstrated that negative superhelicity results in a more active transcription of the immunoglobulin heavy chain gene when only TBP, TFIIB and RNA polymerase II are present [38].

If lack of topoisomerases affects TBP binding, one may speculate, how reinitiation can occur in *gal80Δtop1Δtop2* ^*ts*^ cells. In these cells, TBP is already bound to the DNA template before cells are exposed to the positive supercoiling, which follows the transfer of cells to the restrictive temperature for *top2* ^*ts*^, and although TBP binding may be sensitive to DNA superhelicity, its dissociation may not necessarily be affected. Alternatively, the transient negative supercoiling generated due to polymerase movement during elongation could maintain binding of TBP, as has been suggested earlier [39].

In conclusion, we show that topoisomerase activity is essential for *GAL* gene activation, being required for TBP binding downstream of nucleosome eviction. This is in contrast to our earlier finding with the *PHO5* inducible gene system, where topoisomerase activity was found to be required for binding of the transcription factor Pho4 prior to nucleosome eviction [15]. Topoisomerases can therefore influence different steps in the activation of inducible genes both upstream and downstream of promoter nucleosome clearance. Due to the multitude of protein-DNA interactions required for activation of inducible genes, these genes are thus highly vulnerable to changes in promoter topology.
